# Commentary: Gut microbiota and its derived SCFAs regulate the HPGA to reverse obesity-induced precocious puberty in female rats

**DOI:** 10.3389/fendo.2023.1121124

**Published:** 2023-01-25

**Authors:** Danchun Chen

**Affiliations:** Department of Pediatrics, The Third Affiliated Hospital, Sun Yat-sen University, Guangzhou, China

**Keywords:** obesity, central precocious puberty, high fat diet, SCFA, HPGA

## Introduction

Central precocious puberty occurs when the hypothalamic–pituitary–gonadal axis (HPGA) activates early, with unknown cause in most girls (1). Notably, children with obesity have more chance of precocious puberty (2–8). Although intestinal microbiota, as a remote regulator, was involved in the onset of precocious puberty in high- fat diet (HFD) conditions (9–12), and microbiota- derived SCFAs (short-chain fatty acids) have been characterized as multifunctional molecules impacting human physiology (13), detailed mechanisms remain unclear. Here, Wang et al. found that the reduced microbiota- derived SCFA was accounting for the HFD-induced precocious puberty in female rats (14).

## Findings

The authors (14) firstly confirmed that treatment of HFD after weaning induced obesity and precocious puberty in female rats and upregulated HPGA gene expressions, including Kiss1 (kisspeptin-1), GPR54 (G protein-coupled receptor 54, Kiss1R), GnRH (gonadotropin-releasing hormone), Erα (estrogen receptor α) in the hypothalamus, GnRHR (gonadotropin-releasing hormone receptor) in the pituitary, and LHR (luteinizing receptor), FSHR (follicle-stimulating hormone receptor), and Erα in the ovary. In view of flora, the authors found that HFD significantly changed the composition of intestinal microbiota, with increased levels of Bacteroidota, Proteobacteria, and Verrucomicrobiota but decreased levels of Firmicutes. More importantly, they found that HFD reduced SCFAs in the feces of rats. Moreover, dairy-added SCFAs reversed the obesity-induced female precocious puberty and HPGA key gene expressions. The authors also found that SCFA regulated GnRH release, associated with the inhibited GPR54-PKC-ERK1/2 pathway in the hypothalamus.

## Novelty and contributions

One of the most important innovations of this study is that the authors identified SCFAs as key mediators of HFD-induced precocious puberty in female rats (14). In addition to providing energy, intestinal SCFAs have been previously recognized to regulate metabolism (13, 15, 16), mucosal function (17), immunity (18), and behaviors (19), while the potential regulation of precocious puberty is underappreciated. The authors found that dairy SCFAs can reverse the HFD-induced precocious puberty, through regulating HPGA functions (14). This finding demonstrated the novel function of SCFAs in the regulation of hypothalamus function. In addition, this study added critical knowledge by identifying SCFA as a messenger mediating the gut–endocrine system remote interactions.

Another important finding of this study is that it explained the mechanism of obesity-induced precocious puberty. Although obesity was found as a risk factor for precocious puberty, and fat-conditioned microbiota is found as an important mediator of this axis, the molecular mechanisms remain elusive. By reconstitution of SCFAs in the HFD, this study found that SCFAs reversed the high fat- induced precocious puberty and the key factors of the HPGA. Hence, the study dissected the new molecular mechanism of obesity- mediated precocious puberty, linking obesity and sexual development.

## Potential applications

The golden standard treatment of central precocious puberty is the gonadotropin-releasing hormone agonist (GnRHa), with high efficacy and safety (20, 21). The mechanism of the GnRHa was recognized as suppressing the HPGA and stabilizing pubertal progression (21). However, the treatment has side effects including growth deceleration (22, 23). Thus, there need investigations about the strategies for the medications for the precocious puberty.

There are human studies focusing on the effect of microbiota and their production of SCFAs on glucose and insulin sensitivity (24). Specifically, fiber administration, which facilitated the SCFA- producing microbiota, has been found to favor the outcomes for type 2 diabetes patients (25). In addition, prebiotic functional foods are able to supply SCFAs in treating a variety of disorders including obesity (26). These all give clues for SCFA-involved potential prevention or intervention for precocious puberty.

## Discussion

In this study, Wang et al. found that microbiota-derived SCFAs reversed the high-fat- induced obesity- mediated precocious puberty, with mechanisms of impact on the HPGA (14). Hence, a novel remote brake (gut microbiota-SCFA) of precocious puberty was characterized. Future investigations of this braking system are needed for both mechanistic and therapeutic views. Mechanistically, the kinetics and spatial activities of SCFAs need further investigation, which include the incorporation, metabolism, transportation, and transformation. Importantly, the activities of SCFA and downstream signaling pathways remained to be further clarified, either in the intestine or in the hypothalamus or other compartments of the HPGA. In a therapeutic view, attention should be paid to the strategies of administration of SCFA, related drugs, or prebiotics, with extensive diagnostics and detailed analysis of the outcome of treatment. Furthermore, combined treatment would be encouraged to maximize the effectiveness but minimize the side effects of the drugs. Collectively, Wang et al. found a novel mechanism of obesity- mediated precocious puberty, which provides significant knowledge and potential intervention for imbalanced sexual maturation.

## Author contributions

The author confirms being the sole contributor of this work and has approved it for publication.
